# A presumptive diagnosis of feline hyperesthesia syndrome due to stressful human-related condition: case report

**DOI:** 10.1007/s11259-026-11255-8

**Published:** 2026-05-08

**Authors:** Reiner Silveira de Moraes, Milena da Silva Vieira Ribeiro, Lavínia Laís Corrêa, Hortência Purcena da Guimarães Silveira, Raquel Loren dos Reis Paludo, Cecília Nunes Moreira, Dirceu Guilherme de Souza Ramos, Ísis Assis Braga

**Affiliations:** 1 Department of Veterinary Clinics, School of Veterinary Medicine and Animal Science, São Paulo State University (UNESP), Botucatu, São Paulo Brazil; 2 University Center of Mineiros (UNIFIMES), Mineiros, Goiás Brazil; 3 Graduate Program in Bioscience and One Health, Institute of Agricultural Sciences, Federal University of Jataí, Goiás, Brazil

**Keywords:** Abnormal behavior, Lumbar fasciculation, Multimodal environmental modification, Tail chasing

## Abstract

Feline hyperesthesia syndrome (FHS) is a complex and poorly understood condition characterized by episodic behavioral disturbances, often associated with environmental, neurological, and psychogenic factors. This case report describes the clinical presentation, diagnostic approach, and management of a 43-month-old spayed mixed-breed female cat with a one-year history of behavioral alterations, including lumbar tremors, tail chasing, and sudden aggressive episodes. The onset of clinical signs was temporally associated with a significant environmental change, namely the birth of the owner’s child, which may have acted as a potential stressor; however, a causal relationship cannot be established. Physical examination and hematological evaluation revealed no abnormalities, allowing the exclusion of systemic and dermatological diseases. Due to financial limitations, advanced neurological assessment and imaging investigations were not performed, and a presumptive diagnosis of FHS was established based on clinical history and exclusion criteria. Management was primarily based on multimodal environmental modification (MEMO) strategies aimed at reducing stress and improving behavioral welfare, with adjunctive use of a homeopathic formulation (Anizen^®^). Follow-up evaluations demonstrated a reduction in the frequency and severity of episodes, decreasing from daily occurrences to one or two mild episodes per month, along with improvement in social behavior. This case highlights the importance of environmental and behavioral factors in the clinical expression of FHS and supports the role of structured environmental management as a central component of therapy. Despite the inherent diagnostic limitations, improvement in quality of life remains the primary therapeutic goal, emphasizing the relevance of individualized, multimodal approaches in affected cats.

**Supplementary Information:**

The online version contains supplementary material available at 10.1007/s11259-026-11255-8.

## Background

Feline hyperesthesia syndrome (FHS), also known as “rolling skin disease,” is a complex behavioral condition affecting cats, represented by an exaggerated response to sensory stimuli and compulsive behaviors (Pauciulo et al. 2025). Affected animals exhibit exaggerated reactions to touch, such as muscle contractions along the spine, excessive licking or biting of their own skin, as well as episodes of hyperactivity and aggression (Tuttle 1980; Ciribassi 2009; Munana 2016). These manifestations can be intense and unpredictable, complicating the lives of both the animal and its caregiver. Observations of these behaviors are often sporadic, with periods of normalcy interspersed with episodes of altered behavior (Messa et al. 2020; Schimanski et al. 2023).

The etiology of FHS is not fully understood; however, genetic, environmental, and psychological factors are believed to play a significant role in the development and exacerbation of clinical signs (Ciribassi 2009; Lilly and Siracusa 2024). Chronic stress associated with changes in the home environment, introduction of new animals, or alterations in the animal’s routine can trigger FHS, particularly in psychologically sensitive cats (Ciribassi 2009; Rusbridge 2024). The interplay between behavioral factors, genetic predisposition, and comorbid conditions makes the management of FHS a multifaceted challenge, requiring an approach that integrates environmental modifications, behavioral therapies, and, in some cases, pharmacological interventions (Avril et al. 2025).

Affected cats often display pronounced discomfort during an FHS episode, contributing to the difficulty in managing the condition, as these episodes may arise abruptly (Pauciulo et al. 2025). In certain scenarios, cats appear “hypervigilant” and may present with pupil dilation and excessive vocalization, reflecting a heightened state of sensory arousal (Ciribassi 2009; Rusbridge 2020). Furthermore, clinical variation among affected individuals complicates the recognition and understanding of FHS, making it a true clinical challenge (Avril et al. 2025).

In cases of FHS, differential diagnoses are essential to exclude other conditions that may mimic the clinical signs. This requires laboratory testing, evaluation of the animal’s history, dermatological, neurological, and imaging examinations. Equally important are exclusion tests for diseases such as allergic and hypersensitivity disorders, fungal and bacterial dermatitis, parasitic infections, epilepsy, and neurological or endocrine dysfunctions, which may contribute to establishing the diagnosis of the syndrome (Batle et al. 2019). In some cases, the diagnosis is therapeutic, achieved through intervention with modulatory drugs, environmental modifications aimed at stress reduction, or a combination of both (Virga 2003; Mandigers and Bergknut 2016).

Given these considerations, this case report aimed to present a presumptive diagnosis of FHS, focusing on the identification of contributing stressors and the management strategies employed to promote the animal’s welfare.

## Case description

A 43-month-old spayed mixed-breed female cat was presented for veterinary evaluation due to a history of abrupt temperament changes and behavioral alterations, including lumbar tremors (Supplementary File 1), tail chasing (Supplementary File 2), and episodes of sudden aggression. These episodes occurred daily during crisis periods, predominantly in the morning and late afternoon, and were associated with apparent discomfort, although initially perceived by the owner as transient. Due to the persistence and progression of these signs, the animal was referred for further investigation.

According to the anamnesis, the onset of clinical signs occurred approximately one year prior to presentation and coincided with the birth of the owner’s first child. During this period, the cat began to exhibit rejection behaviors toward the child, as well as inappropriate urination in the child’s crib, suggesting a possible association between environmental stress and the emergence of behavioral disturbances. The animal had no outdoor access or contact with other animals and was maintained on a commercial diet formulated for neutered cats. Preventive care, including vaccination and antiparasitic prophylaxis with selamectin (Revolution^®^), was reported to be up to date.

**Fig. 1:**
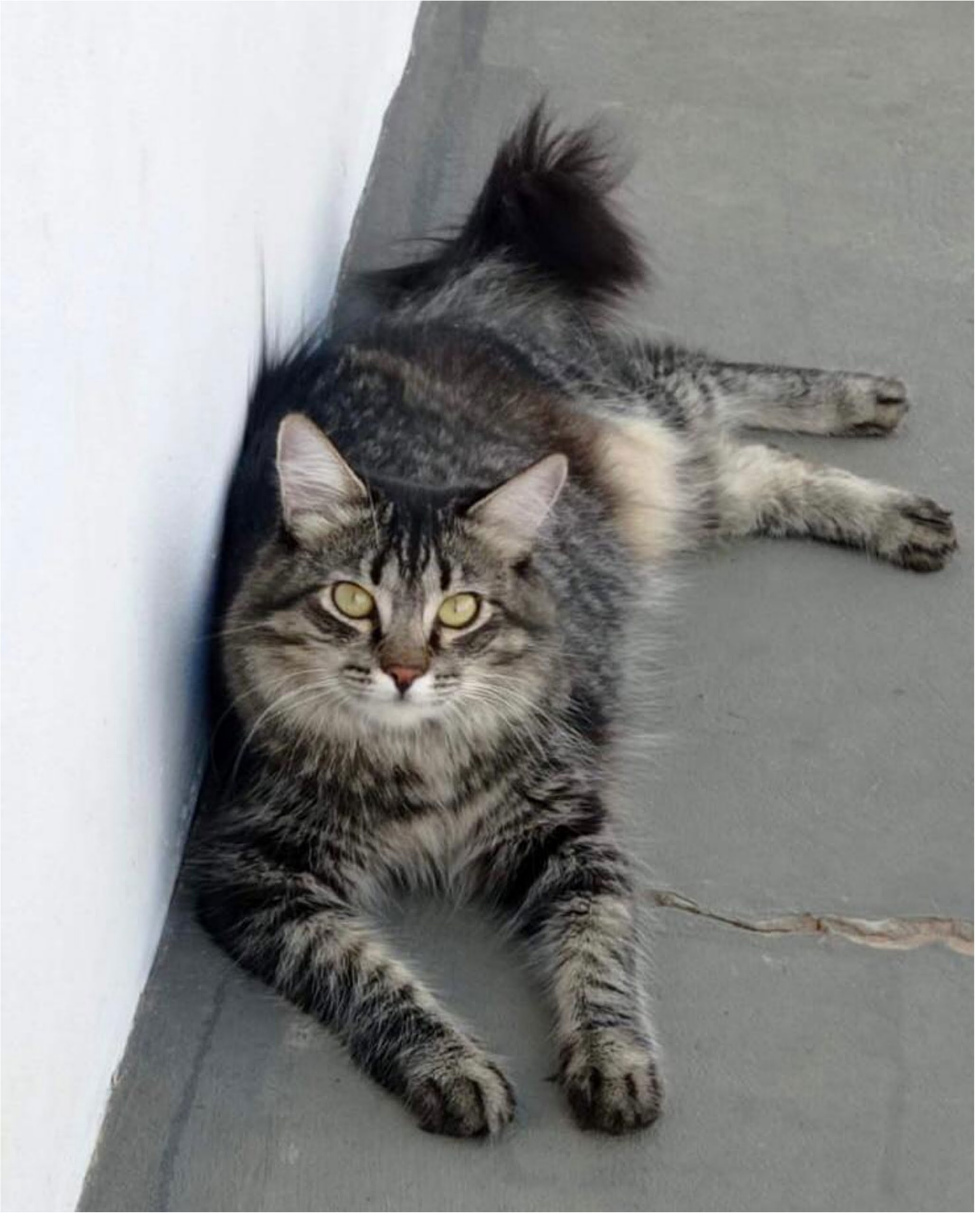
Adult female domestic mixed-breed cat with a presumptive diagnosis of feline hyperesthesia syndrome (FHS) following a stressful event. In this image, the animal is observed outside of a crisis, with no dermatological abnormalities

At presentation, physical examination revealed normal clinical parameters, including a heart rate of 128 beats per minute, respiratory rate of 25 movements per minute, and body temperature of 38.2 °C, along with adequate hydration and a strong, regular femoral pulse. These parameters were within the physiological limits expected for the species (Quimby et al. 2011). No dermatological abnormalities, such as alopecia or cutaneous lesions, were observed, which contributed to excluding dermatological conditions as a potential cause of the behavioral signs (Fig. 1).

A complete hematological evaluation was subsequently performed, revealing erythrocyte, leukocyte, and platelet values within normal reference ranges (Brooks et al. 2022). The absence of hematological alterations suggested that infectious, inflammatory, or systemic disorders were unlikely to be contributing to the clinical presentation. In order to perform a complete investigation of the clinical condition of the animal, diagnostic procedures, such as detailed physical neurological evaluation was performed. In addition, in order to evaluate the nervous system through brain and spinal cord structures, imaging examinations based on magnetic resonance imaging (MRI) and computed tomography (CT) were indicated. However, owner financial restrains did not allow the investigation for neurological disturbances, making the presumptive diagnosis of FHS to be considered and then, the appropriate management to be implemented.

Based on the animal’s history, clinical signs, and the exclusion of systemic and dermatological diseases, a presumptive diagnosis of FHS was established. Treatment was primarily started with the implementation of environmental management strategies, including the provision of physical stimuli through toys, scratching posts, and vertical spaces, as well as increased social interaction and play sessions to promote mental stimulation and reduce stress levels. As an adjuvant therapy, a homeopathic formulation (Anizen^®^), administered every eight hours for a period of 30 days was added.

At the one-month follow-up, the owner reported a moderate reduction in the frequency of episodes, which decreased to approximately two moderate crises per month. However, inconsistent administration of the medication was noted due to difficulties in maintaining the prescribed schedule and the cat’s reluctance to accept treatment, which may have limited the therapeutic response. Consequently, strict adherence to the treatment protocol was emphasized, along with continued reinforcement of environmental enrichment measures.

**Fig. 2:**
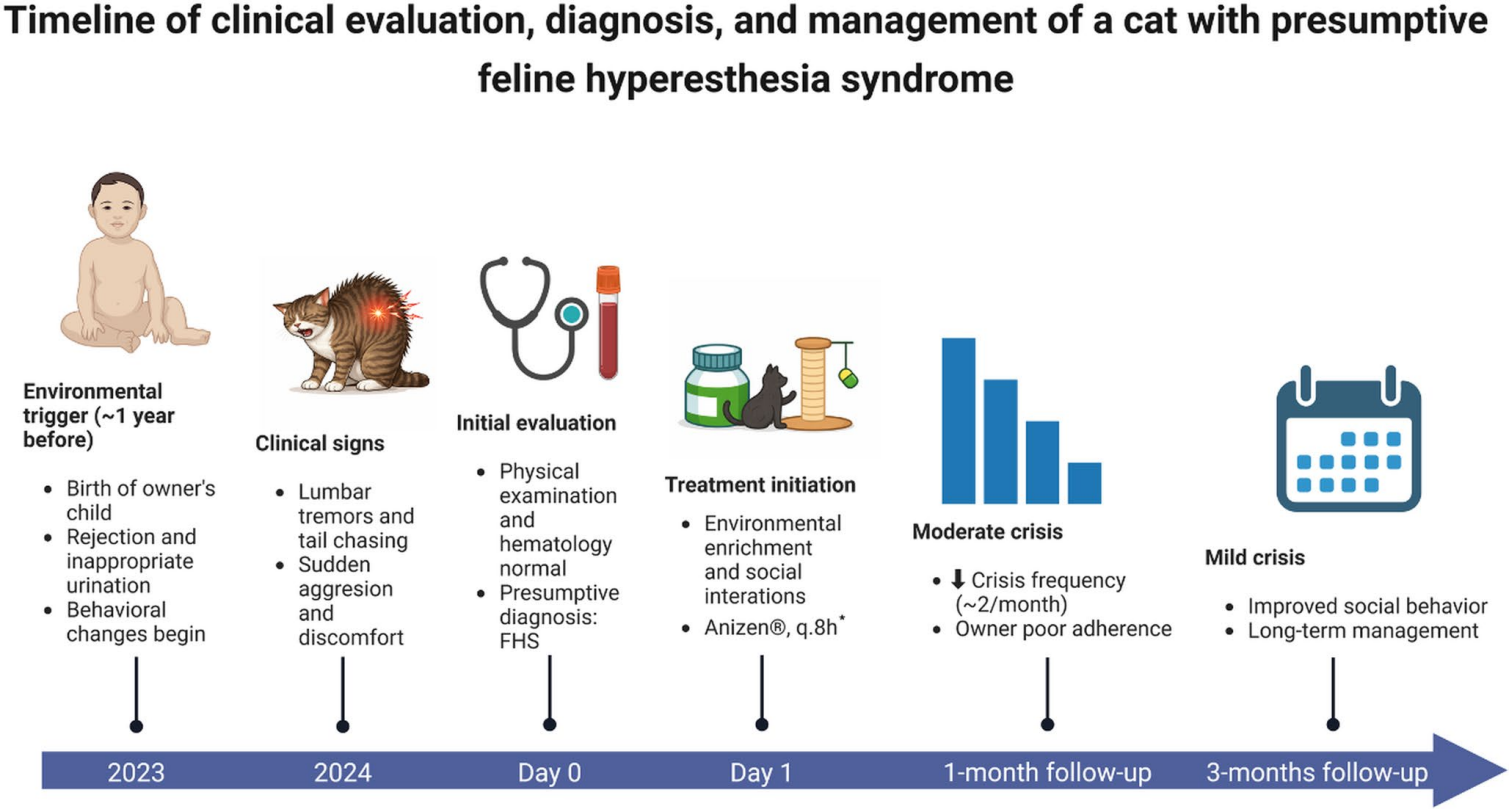
Timeline of the events. *It was implemented as adjuvant treatment for 30 days. The basis of the treatment was the Multimodal Environmental Modification (MEMO)

Three months after the initial consultation, the cat continued to present one or two mild episodes per month. Although the frequency of crises remained relatively stable, a marked improvement in social behavior was observed up to recent days (two years later), with the animal becoming more receptive and interactive within the household environment (Supplementary File 3). This improvement was attributed primarily to enhanced environmental management and increased owner engagement, highlighting the importance of a multimodal approach in the management of FHS. Based on the observed clinical evolution, the owner was oriented to maintain long-term treatment, combining continuous medication administration with environmental and behavioral interventions aimed at sustaining the animal’s quality of life. The timeline of the events is shown in Fig. 2.

## Discussion

Feline hyperesthesia syndrome (FHS) represents a diagnostic challenge in veterinary medicine due to the absence of specific tests capable of definitely confirming the condition (Kay 2025). Ideally, the diagnosis relies on the identification of characteristic clinical signs combined with the systematic exclusion of differential diagnosis (Lorimier 2009; Batle et al. 2019). However, as observed in the cat described in this case report, the presumptive diagnosis may be considered.

In the present case, the absence of abnormalities in laboratory tests, including complete blood count, serum biochemistry, and urinalysis, allowed the exclusion of systemic imbalances such as renal or hepatic dysfunction (Pauciulo et al. 2025). Similarly, dermatological evaluation, including skin scraping and fungal culture, revealed no alterations, and no dietary changes were identified that could suggest food hypersensitivity. These findings support the exclusion of conditions that may induce pruritus and compulsive behaviors, such as excessive grooming or self-mutilation (Batle et al. 2019; Avril et al. 2025). Additionally, although a direct causal relationship cannot be established, the presence of behavioral changes temporally associated with an identifiable stressor—namely, the introduction of a new family member—suggests that environmental stress may have contributed to the manifestation of clinical signs, supporting the presumptive diagnosis of FHS (Messa et al. 2020; Rusbridge 2020).

Nevertheless, it is important to emphasize that a detailed neurological examination performed by a specialist was not conducted, which limits precise neuroanatomical localization. Furthermore, advanced imaging techniques, such as magnetic resonance imaging (MRI) and computed tomography (CT), were not performed due to owner financial constraints. These modalities are essential to evaluate not only the brain but also the spinal cord, allowing exclusion of structural, inflammatory, or neoplastic conditions that may mimic FHS (March et al. 1999; Schimanski et al. 2023). Electroencephalography may also be useful to exclude neurological disorders such as epilepsy, which can present with similar clinical manifestations (Álvarez and Arias 2021). Therefore, the diagnosis in this case must be interpreted with caution.

In the absence of confirmatory diagnostic tools, a therapeutic approach may contribute to the clinical reasoning process. In this case, the reduction of clinical signs following the implementation of multimodal environmental modification (MEMO) such as the introduction of environmental enrichment items such as toys, scratching posts, and shelves, along with increased owner–animal interaction supports the behavioral and stress-related component of the syndrome. Environmental management strategies, including appropriate management of the space, water and diet, litter boxes, play opportunities, and increased owner–animal interaction, are considered essential for reducing stress and improving behavioral alterations in cats (Westropp et al. 2019).

Although Anizen^®^ was included as part of the adjuvant therapeutic protocol, its efficacy in cats with FHS remains poorly documented (Valle et al. 2024), and its contribution to the observed improvement cannot be definitively established. Integrative approaches, such as the use of essential oils, floral essences, and phytotherapeutic agents, have been explored with variable outcomes, generally as adjunctive therapies rather than primary treatments (Uccheddu et al. 2023). Furthermore, pharmacological interventions, including anticonvulsants and anxiolytics, do not consistently eliminate clinical episodes, reflecting the complexity of this condition (Pauciulo et al. 2025).

Finally, caregiver compliance played a critical role in the clinical outcome, as adherence to environmental modifications and careful monitoring of behavioral changes are essential for therapeutic success (Casey and Bradshaw 2008; Pauciulo et al. 2025). Despite the inherent limitations of this case, the clinical evolution observed reinforces the importance of a structured diagnostic approach and highlights the relevance of environmental management in the control of FHS-related clinical signs.

## Conclusion

Feline hyperesthesia syndrome represents a significant clinical challenge in veterinary practice due to its complex nature and the absence of specific diagnostic tests. In the present case, a multidisciplinary management approach incorporating environmental modification (MEMO) and supportive therapy supported a clinically reasoned presumptive diagnosis, despite the absence of advanced imaging and specialized neurological evaluation. The onset of clinical signs was temporally associated with the introduction of a new family member, which may have acted as a potential stressor; however, a direct causal relationship cannot be established. Although complete control of clinical signs—such as lumbar tremors and tail chasing—may be difficult to achieve, improvement in the animal’s quality of life remains the primary therapeutic goal. Further research is essential to better elucidate the underlying mechanisms of FHS and to support the development of more effective diagnostic and therapeutic strategies.

## Supplementary Information

Below is the link to the electronic supplementary material.

Supplementary Material 1

Supplementary Material 2

Supplementary Material 3

## Data Availability

The datasets generated during and/or analyzed during the current study are available from the corresponding author on reasonable request.
